# Long non-coding RNA DLX6-AS1 facilitates bladder cancer progression through modulating miR-195-5p/VEGFA signaling pathway

**DOI:** 10.18632/aging.103374

**Published:** 2020-08-03

**Authors:** Hengbing Wang, Xiaobing Niu, Hesong Jiang, Fei Mao, Bing Zhong, Xi Jiang, Guangbo Fu

**Affiliations:** 1 Department of Urology, The Affiliated Huaian No.1 People's Hospital of Nanjing Medical University, Huai'an 223300, Jiangsu Province, China

**Keywords:** bladder cancer, DLX6-AS1, lncRNA, miR-195-5p, VEGFA

## Abstract

In this study, we aim at investigating the expression and regulation role of long non-coding RNA (lncRNA) DLX6-AS1 in bladder cancer (BC). DLX6-AS1 was highly expressed in BC tissues and significant negative correlation with the 5-year survival in the BC patients. The results showed that the proliferation, migration and invasion activities of BC cells were promoted by DLX6-AS1 overexpression, while cell apoptosis was repressed. However, knockdown DLX6-AS1 presented an pposite regulatory effect, and DLX6-AS1 knockdown delayed tumor *in vivo*. The potential target of DLX6-AS1 in BC was predicted and verified by RIP, RNA pull-down, and dual-luciferase reporter assays as miR-195-5p. The results showed that miR-195-5p was down-regulated in BC tissues, the expression of which was significantly negative correlated with DLX6-AS1 expression. In addition, the results also showed that miR-195-5p targeted and down-regulated the VEGFA. Knockdown of DLX6-AS1 up-regulated miR-195-5p expression and down-regulated VEGFA expression. Moreover, down-regulation of VEGFA expression caused by DLX6-AS1 inhibited phosphorylation of Raf-1, MEK1/2, and ERK1/2, while miR-195-5p inhibitors abolished the effect of silencing DLX6-AS1 expression. Our study demonstrated that DLX6-AS1 played an oncogenic role in BC through miR-195-5p-mediated VEGFA/Ras/Raf/MEK/ERK pathway.

## INTRODUCTION

Bladder cancer (BC) is a malignant tumor that occurs in the bladder mucosa, and BC is also the most common malignant tumor in the urinary system. According to the updated cancer statistics in 2017, there were 79 030 cases and 16 870 deaths of urinary BC were estimated which is the sixth most common cancer and the eighth leading cause of death due to cancer in the United States [1]. The latest cancer statistics in 2015 for China showed that there were about 80 500 new cases of BC, including 62 100 males and 18 400 females were diagnosed. The incidence of BC has surpassed prostate cancer and become the first urinary malignancies in male [2]. Although most patients have non-muscle invasive carcinoma at the initial diagnosis, these tumors are prone to recurrence, infiltration and drug resistance after treatment, which becomes a huge challenge for clinical treatment. In recent years, the survival rate of cancer patients was significantly improved. However, The 5-year survival rate of BC is still at a low level. Until recently, the treatment for BC had seen little progress [3]. The pathobiological mechanisms of BC are still unclear. Therefore, exploring biomarkers or new therapeutic targets will facilitate the research of BC treatments.

Long non-coding RNA (lncRNA) is a subgroup of non-coding RNAs consisting of more than 200 nucleotides originally considered to be by-products of RNA polymerase II transcription and do not have any biological function [4]. Recent studies show that lncRNA participates in many important regulatory processes in terms of epigenetic, transcriptional and post-transcriptional regulation [5] LncRNA is becoming one of the hotspots in the field of oncology. A number of studies demonstrate that abnormal expressions of lncRNAs exist in various kinds of malignant tumors [5, 6]. In recent years, the role of lncRNA in tumorigenesis, metastasis, and progression of BC has also attracted extensive concern [7, 8]. Studies have reported that several lncRNAs such as UCA1, MALAT1, ncRNA, GAS5, and H19 are involved in the development and progression of BC [9, 10]. Several studies reported that DLX6-AS1 was abnormally expressed in some malignancies and play a crucial role in cancer occurrence and progression [11]. DLX6-AS1 is located on human chromosome 7q21.3 and has been shown to be over-expressed in hepatic carcinoma, lung cancer, pancreatic cancer, and renal cell carcinoma and shows carcinogenesis effect in the development of these cancers [11, 12]. However, the biological role of DLX6-AS1 in BC has not been clearly studied, and its biological function and regulatory role still need to be further investigated.

In this study, we investigated the expression of DLX6-AS1 in BC clinical samples and its regulatory effect and potential mechanisms in BC development and progression. Our results suggested that DLX6-AS1 was up-regulated in BC tissues and BC cell lines, and increased expression of DLX6-AS1 promoted the proliferation, migration, and invasion activities of BC cells *in vitro* and promoted tumor growth *in vivo*. Inhibition of DLX6-AS1 expression showed an anti-tumor effect. The underlying regulating mechanism of DLX6-AS1 in BC was also investigated. Overall, our study indicates that DLX6-AS1 plays a regulatory role in development and metastasis of BC through miR-195-5p-mediated VEGFA/Ras/Raf/MEK/ERK signaling pathway, and the DLX6-AS1 may be a potential biomarker and target for BC.

## RESULTS

### DLX6-AS1 is up-regulated in BC tissues and cells

**Figure 1:**
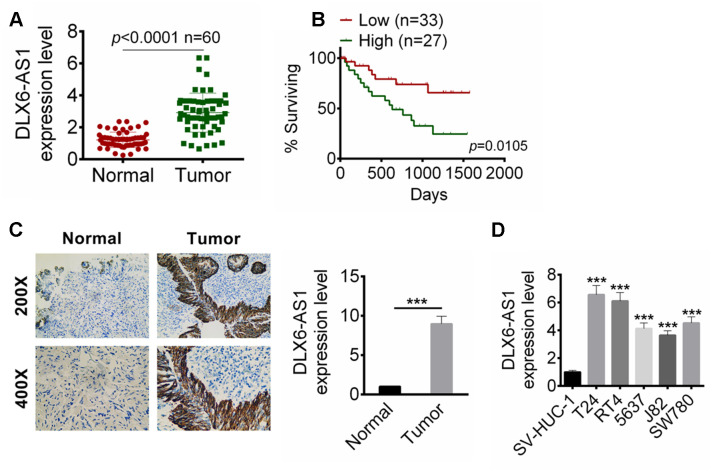
**DLX6-AS1 was up-regulated in BC tissues and cell lines.** (**A**) The DLX6-AS1 expression in 60 cases of BC tissues and adjacent normal tissues were determined by qRT-PCR. (**B**) The relationship of 5 year survival rate of the BC patients with high (n=27) or low (n=33) DLX6-AS1 expression were analyzed by Kaplan-Meier’s method and the log-rank test. (**C**) The expression of DLX6-AS1 in BC tissues and normal tissues was determined by ISH assay. (**D**) The mRNA expression level of DLX6-AS1 in a human bladder epithelium immortalized cell line (SV-HUC-1) and five BC cell lines (T24, RT4, 5637, J82, and SW780) was determined by qRT-PCR. The DLX6-AS1 was highly expressed in BC cells. Data were expressed as the mean ± SD. ***P < 0.001 vs SV-HUC-1.

We first examined the expression of DLX6-AS1 in BC tissues and adjacent normal tissue samples from 60 BC patients by qRT-PCR. As shown in Figure 1A, the expression level of DLX6-AS1 in BC tissues was significantly higher than that in normal tissues (n=60, *P* < 0.0001). Then, these BC patients were divided into high DLX6-AS1 group (≥ median, n = 27) and low DLX6-AS1 group (<median, n = 33) according to the expression level of DLX6-AS1. The 5-year survival of these patients was recorded. As shown in Figure 1B, the low DLX6-AS1 expression patients showed a better survival rate compared with that of high DLX6-AS1 expression (*P*=0.0105). The in situ hybridization (ISH) assay also demonstrated that DLX6-AS1 was highly expressed in BC tissues (Figure 1C). In addition, we evaluated the expression of DLX6-AS1 in a human bladder epithelium immortalized cells (SV-HUC-1) and several BC cell lines (T24, RT4, 5637, J82, and SW780). The results suggested that the DLX6-AS1 was significantly up-regulated in the BC cell lines (T24, RT4, 5637, J82, and SW780) than that of SV-HUC-1 cells (more than 4-fold, Figure 1D). T24 and RT4 cells showed the higher expression of DLX6-AS1 than other BC cell lines, and were selected for the further studies.

### DLX6-AS1 promotes BC cell proliferation in vitro and growth in vivo

**Figure 2:**
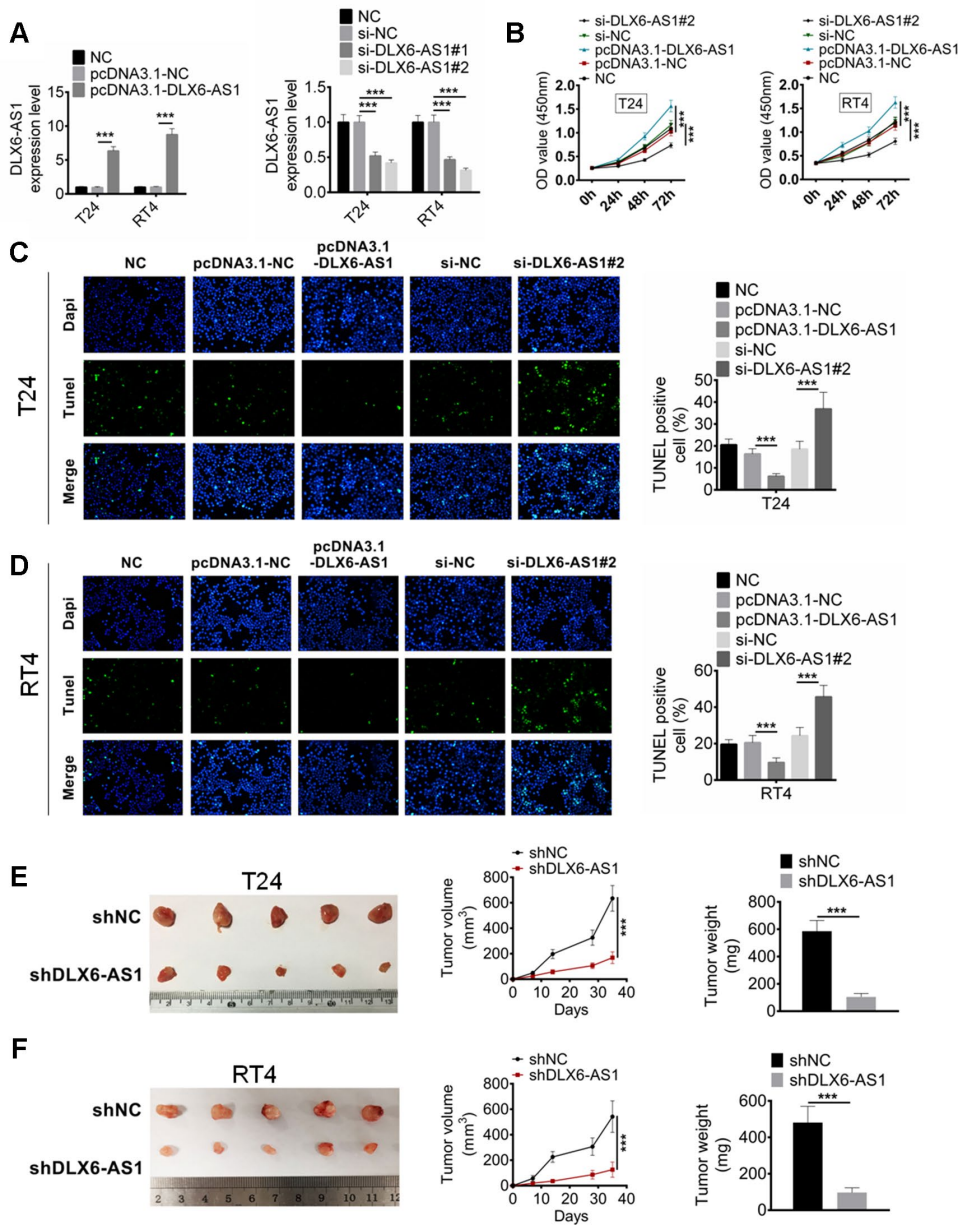
**DLX6-AS1 promotes proliferation and inhibits apoptosis of BC cells *in vitro* and *in vivo*.** (**A**) The expression of DLX6-AS1 was over-expressed by pcDNA3.1-DLX6-AS1 or down-regulated by sh-DLX6-AS1 in T24 and RT4 cells, and the transfection efficiency was identified by qRT-PCR. (**B**) Cell proliferation of T24 and RT4 cells with pcDNA3.1-DLX6-AS1 or sh-DLX6-AS1 transfection was determined by CCK-8 assay. (**C**, **D**) The apoptosis of T24 and RT4 cells with pcDNA3.1-DLX6-AS1 or sh-DLX6-AS1 transfection was determined by TUNEL staining assay. (**E**, **F**) A subcutaneous BC tumor model (n=6/group) was established by using T24 and RT4 cells to evaluate the anti-tumor effect of shDLX6-AS1, and tumor volume and weight were calculated. Data were expressed as the mean ± SD. ***P < 0.001.

In order to investigate the biological role of DLX6-AS1 in BC, the expression vector carried DLX6-AS1 or siRNA against DLX6-AS1 was constructed and transfected into the T24 and RT4 cells to over-express or knockdown the DLX6-AS1 expression. As shown in Figure 2A, pcDNA3.1-DLX6-AS1 and sh-DLX6-AS1 successfully up-regulated and down-regulated the expression of DLX6-AS1 in T24 and RT4 cells compared with the NC and siNC group, respectively. Subsequently, the effects of pcDNA3.1-DLX6-AS1 and sh-DLX6-AS1 on BC cells proliferation and apoptosis were examined. As shown in Figure 2B, pcDNA3.1-DLX6-AS1 obviously promoted the proliferation of T24 and RT4 cells. However, this stimulative effect was inhibited by sh-DLX6-AS1 significantly. The apoptosis effect of DLX6-AS1 on the BC cell was detected by TUNEL staining assay. The results suggested that sh-DLX6-AS1 increased the number of apoptotic cells, whereas pcDNA3.1-DLX6-AS1 remarkably inhibited BC cells apoptosis (Figure 2C, 2D). Moreover, the effect of DLX6-AS1 knockdown on BC tumor growth was also investigated by a subcutaneous xenograft mouse model. We found that sh-DLX6-AS1 drastically inhibited tumorigenicity of BC cells (Supplementary Figure 1A), and sh-DLX6-AS1 decreased the tumor volume and weight compared with the control group mice (Figure 2E, 2F). The sh-DLX6-AS1 group mice also presented a lower DLX6-AS1 and Ki67 expression compared with the control group which determined by IHC analysis (S upplementary Figure 1E).

### DLX6-AS1 increases the migration and invasion activities BC cells

**Figure 3:**
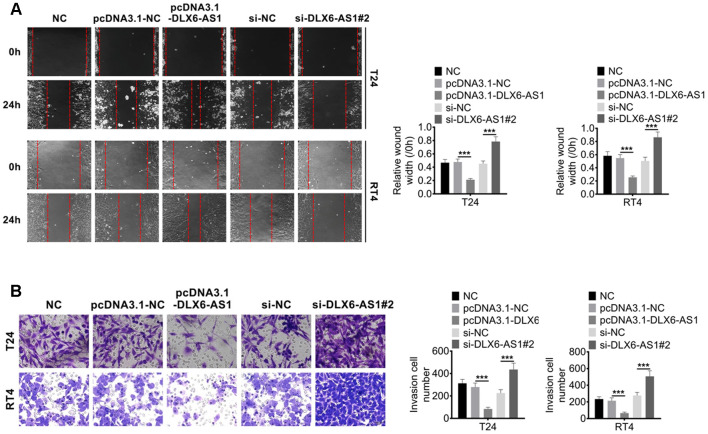
**DLX6-AS1 promotes migration and invasion of BC cells.** (**A**) The migration of BC cells with pcDNA3.1-DLX6-AS1 or sh-DLX6-AS1 transfection was determined by wound healing assay (**B**) The invasion of BC cells with pcDNA3.1-DLX6-AS1 or sh-DLX6-AS1 transfection was determined by Transwell chamber assay. Data were expressed as the mean ± SD, n = 3. ***P < 0.001.

Our study demonstrated that DLX6-AS1 over-expression promoted the proliferation activity of BC cells *in vitro*. We subsequently investigated the effect of DLX6-AS1 on BC cell migration and invasion activity. As shown in Figure 3A, the control groups of NC, pcDNA3.1-NC and sh-NC showed a similar relative wound gap width. While, pcDNA3.1-DLX6-AS1 accelerated the healing of the wound gap and sh-DLX6-AS1 inhibited the wound healing. Furthermore, pcDNA3.1-DLX6-AS1 significantly promoted the ability of BC cells to invade through matrigel, while sh-DLX6-AS1 significantly reduced this invasion behavior (Figure 3B). Therefore, these results suggested that DLX6-AS1 exerted a role in accelerating BC cells migration and invasion.

### DLX6-AS1 targets miR-195-5p

**Figure 4:**
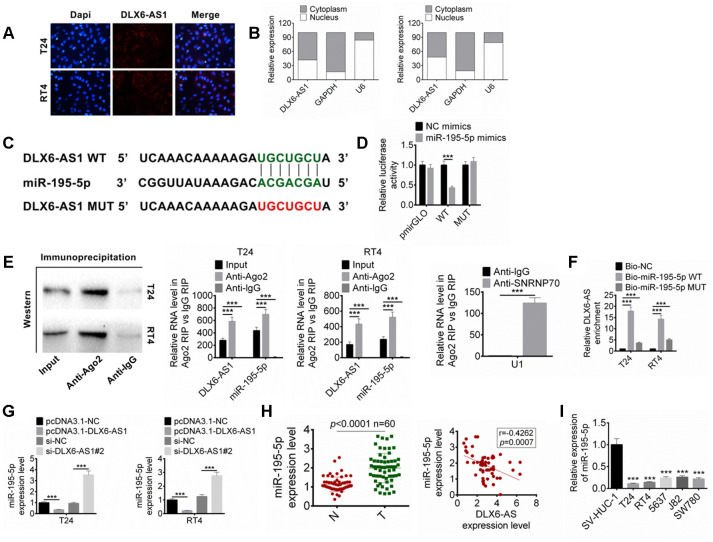
**DLX6-AS1 targets miR-195-5p.** (**A**) The expression of DLX6-AS1 in BC cells was determined by FISH assay. (**B**) The specific location of DLX6-AS1 was identified in BC cells through performing subcellular fractionation assay. (**C**) The binding sites of DLX6-AS1 and miR-195-5p was predicted. (**D**) The binding of miR-195-5p and DLX6-AS1 was verified by dual-luciferase reporter assay. (**E**) The binding of miR-195-5p and DLX6-AS1 was validated by RIP assay. (**F**) The binding of miR-195-5p and DLX6-AS1 was validated by RNA pull down assay. (**G**) The expression of miR-195-5p in BC cells with DLX6-AS1 overexpression or knockdown was determined by qRT-PCR. (**H**) The expression of miR-195-5p in BC tissues and the relationship between DLX6-AS1 and miR-195-5p expression were determined. (**I**) The miR-195-5p expressions in BC cell lines (T24, RT4, 5637, J82, and SW780) and SV-HUC-1 cells by qRT-PCR. Data were expressed as the mean ± SD, n = 3. ***P < 0.001.

The expression and location of DLX6-AS1 in BC cells were detected by FISH and subcellular fractionation assay. The results suggested that the DLX6-AS1 mainly expressed in the cytoplasm (Figure 4A, 4B). We predicted the possible target miRNAs for DLX6-AS1 by bioinformatics analysis, the results showed that DLX6-AS1 might target miR-195-5p. The complementary binding sequence with miR-195-5p and a mutant sequence of DLX6-AS1 were shown in Figure 4C. The dual-luciferase reporter assay exhibited that miR-195-5p mimics significantly reduced the luciferase activity of DLX6-AS1-wt, while had no significant effect on DLX6-AS1-mut, indicating that miR-195-5p can bind to DLX6-AS1 sequence (Figure 4D). In order to verify whether DLX6-AS1 and miR-195-5p bind through the ribonucleoprotein complex of miRNA, RIP assay and Ago2 antibody were used to capture the Ago2 protein and its binding RNA. The results showed that DLX6-AS1 and miR-195-5p were combined with Ago2 (Figure 4E). In addition, their binding was also validated by biotin-labeled RNA pull-down assay. As shown in Figure 4F, a large amount of Bio-miR-195-5p WT was detected in the DLX6-AS1 pull-down pellets compared to the Bio-NC control or the Bio-miR-195-5p MUT group by qRT-PCR.

In addition, our results also suggested that over-expression of DLX6-AS1 significantly decreased miR-195-5p level and knockdown of DLX6-AS1 displayed an opposite effect on BC cells (Figure 4G). So the expression of miR-195-5p in the BC tissues and its relationship with DLX6-AS1 were further studied. The results showed that the expression level of miR-195-5p in BC tissues was significantly lower than that in normal tissues, which was negatively correlated with the expression of DLX6-AS1 (Figure 4H). The expressions of miR-195-5p were also down-regulated in BC cell lines (Figure 4I). These results suggested that DLX6-AS1 may directly bind to miR-195-5p to inhibit its activity.

### MiR-195-5p targets VEGFA and inhibits BC cells proliferation, migration, and invasion

**Figure 5:**
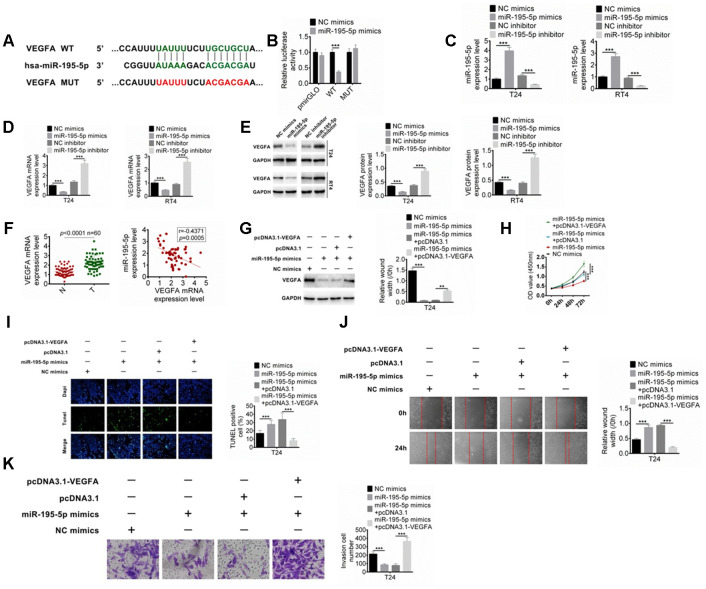
**MiR-195-5p combines with VEGFA.** (**A**) The target of miR-195-5p was predicted by Targetscan. (**B**) The binding of miR-195-5p and VEGFA was verified by dual-luciferase reporter assay. (**C**) BC cells were transfected with miR-195-5p mimics or inhibitors, and the transfection efficiency of miR-195-5p was determined by qRT-PCR. (**D**) The mRNA expression of VEGFA in T24 and RT4 cells with miR-195-5p mimics or miR-195-5p inhibitors was examined by qRT-PCR. (**E**) The protein expression of VEGFA in T24 and RT4 cells were identified by western blot. (**F**) The VEGFA expression in BC tissues and the relationship of VEGFA and miR-195-5p expression were explored. (**G**) The VEGFA protein expression in T24 cells with miR-195-5p mimics transfection, or miR-195-5p mimics+ pcDNA3.1 co-transfection was determined by western blot. (**H**–**K**) Cell proliferation, apoptosis, migration, and invasion of T24 cells were examined by CCK8, Tunel, scratched wound healing and transwell assay, respectively. Data were expressed as the mean ± SD, n = 3. **P < 0.01, ***P < 0.001.

The downstream target for miR-195-5p was also predicted, and VEGFA was predicted as the target of miR-195-5p (Figure 5A). Their binding was determined by dual-luciferase reporter assay. As shown in Figure 5B, miR-195-5p mimics significantly reduced the luciferase activity of DLX6-AS1-wt, while had no significant effect on DLX6-AS1-mut. Furthermore, the effect of miR-195-5p on the VEGFA expression was studied. The expression efficiency of the inhibitor and mimics of miR-195-5p was confirmed in Figure 5C. The results showed that the mRNA and protein expression levels of VEGFA in BC cells were significantly decreased by miR-195-5p mimics and significantly increased by miR-195-5p inhibitor (Figure 5D, 5E). However, pcDNA3.1-VEGFA could abrogate the regulation of miR-195-5p mimics on the VEGFA expression in BC cells (Figure 5F). The expression of VEGFA in the BC tissues and its relationship with miR-195-5p expression were further investigated. The results showed that the expression level of VEGFA in BC tissues was significantly higher than that in normal tissues, which was negatively correlated with the miR-195-5p expression (Figure 5G). Moreover, the role of miR-195-5p and VEGFA in the proliferation, apoptosis, migration, and invasion of BC cells were evaluated. The results showed that up-regulation of miR-195-5p showed anti-tumor effects such as inhibition of proliferation, induction of apoptosis, and inhibition of invasion and migration on BC cells, while these effects were abrogated by the pcDNA3.1-VEGFA co-transfection (Figure 5H–5K). Moreover, we examined the expressions of miR-195-5p and VEGFA in the tumor tissues of the xenograft mice, and the results showed that shDXL6-AS1 significantly promoted the miR-195-5p expression and suppressed the VEGFA expression compared with the shNC group (Supplementary Figure 1B, 1C and 1E). These results suggested that miR-195-5p may inhibit BC cells proliferation and metastasis through targeting VEGFA.

### The miR-195-5p inhibitors rescued the effect of si-DLX6-AS1 on BC cells

**Figure 6:**
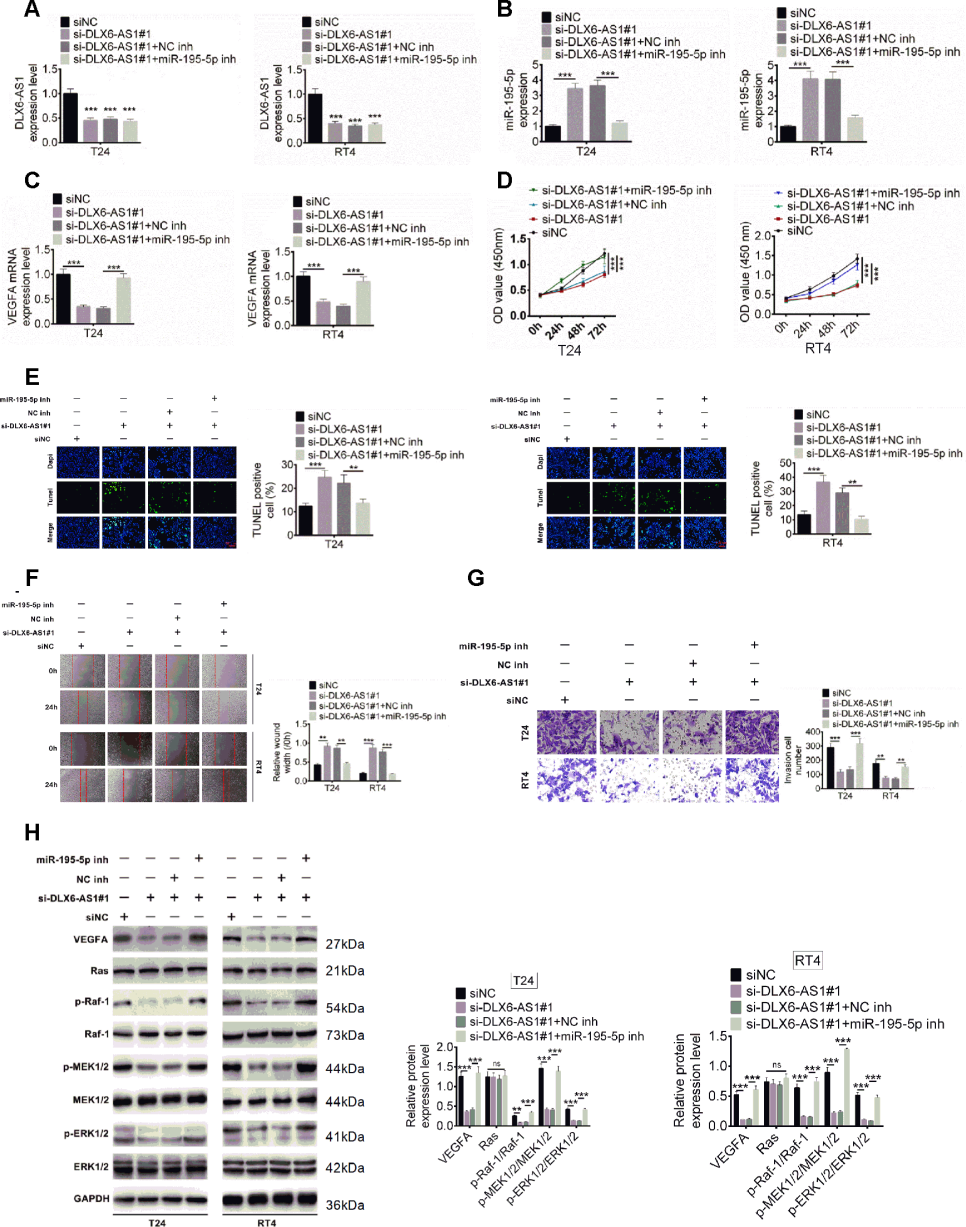
**Effect of DLX6-AS1 and miR-195-5p on proliferation, migration, and invasion of BC cells and phosphorylation of VEGFA, Ras, MEK1/2, ERK1/2.** (**A**–**C**) BC cells were transfected with siNC, siNC + si-DLX6-AS1, si-DLX6-AS1 + NC inhibitor, or si-DLX6-AS1 + miR-195-5p inhibitor. DLX6-AS1 expression (**A**), miR-miR-195-5p expression (**B**), and VEGFA mRNA expression (**C**) were examined by qRT-PCR assay. (**D**) Cell proliferation of BC cells was identified by CCK8 kit. (**E**) Cell apoptosis was examined by TUNEL assay. (**F**) Cell migration was detected by wound healing assay. (**G**) Cell migration was identified by transwell assay. (**H**) The effect of sh-DLX6-AS1 and miR-195-5p inhibitor on protein expression and phosphorylation levels of VEGFA and Ras/ERK signaling molecules was evaluated by western blot. Data were expressed as the mean ± SD, n = 3. *P < 0.05, **P < 0.01, ***P < 0.001.

The underlying molecular mechanisms of DLX6-AS1 and miR-195-5p on BC were further explored. T24 and RT4 cells were transfected with siNC, or si-DLX6-AS1, or si-DLX6-AS1 + NC inh, or si-DLX6-AS1 + miR-195-5p inhibitors, the expressions of DLX6-AS1, miR-195-5p, and VEGFA were evaluated. As shown in Figure 6A–6C, si-DLX6-AS1 significantly increased miR-195-5p expression and decreased VEGFA expression, while miR-195-5p inhibitors increased the VEGFA expression in si-DLX6-AS1-treated cells. In addition, knockdown of DLX6-AS1 inhibited the proliferation, migration, and invasion activity and increased the apoptosis of BC cells (Figure 6D–6G). However, the effect of si-DLX6-AS1 on BC cells were effectively reversed by miR-195-5p inhibitors. Furthermore, we evaluated the regulation of DLX6-AS1 on VEGFA and Ras/ERK signaling pathway. As shown in Figure 6H, knockdown of DLX6-AS1 reduced VEGFA expression significantly but had no significant effect on Ras expression. Knockdown of DLX6-AS1 had no effect on the expression of Raf-1, MEK1/2, and ERK1/2 but significantly inhibited their phosphorylation. However, the effects of si-DLX6-AS1 on the VEGFA/ Ras/Raf/MEK/ERK signaling were abrogated by miR-195-5p inhibitors. These results indicated that DLX6-AS1 promoted BC cells proliferation, migration and invasion activity through regulating miR-195-5p-mediated VEGFA/ Ras/Raf/MEK/ERK signaling pathway.

## DISCUSSION

LncRNAs have multiple biological functions and participate in a lot of physiological and pathological processes of human body [13]. The abnormal expressions of lncRNAs can promote or inhibit the expression of some oncogenes. In recent years, the biological functions of lncRNAs in the development and progression of tumors and its potential value in screening biomarkers for early diagnosis of tumors have attracted much attention [13, 14]. Previous studies reported that DLX6-AS1 was over-expressed in some cancers. In this study, we found that DLX6-AS1 was not only up-regulated in BC tissues, but also highly expressed in BC cell lines such as T24, RT4, 5637, J82, and SW780 cells. DLX6-AS1 showed a lower level in normal tissues and human bladder epithelium cells. We collected the 5-year survival data of enrolled BC patients and analyzed its relationship with DLX6-AS1 expression by Kaplan-Meier’s method. The results showed that the expression of DLX6-AS1 in BC tissues was negatively correlated with the 5-year survival rate of the patients. These results indicates that DLX6-AS1 acts as an oncogene in BC.

In order to illustrate the biological function of DLX6-AS1 in the development of BC more comprehensively, we examined the effect of DLX6-AS1 over-expression or knockdown on biological behaviors of BC cells. Our results showed that over-expression of DLX6-AS1 facilitated cell proliferation activity and inhibited cell apoptosis of BC cells. Knockdown of DLX6-AS1 showed an anti-tumor effect on BC cells. In addition, a subcutaneous xenograft mouse model was established to assess the effect of DLX6-AS1 on tumor growth *in vivo*. We found that knockdown of DLX6-AS1 inhibited BC tumor growth in mice. Furthermore, the effect of DLX6-AS1 knockdown on migration and invasion were also investigated, and the results suggested that DLX6-AS1 silencing inhibited migration and invasion of BC cells. These data indicates that DLX6-ASl promotes BC malignant cell phenotype *in vivo* and *in vitro*.

MicroRNA (miRNA) is a class of endogenous non-coding small RNAs containing 17-25 nucleotides, and it regulates various biological processes such as cell growth, division, metabolism and development [15]. A number of studies report that lncRNA can adsorb specific miRNA by sponge effect or competitively bind miRNA to regulate the binding of endogenous miRNA and its target gene [16, 17]. In this study, we found that the downstream target of DLX6-AS1 is miR-195-5p. It was reported that miR-195-5p acted as a tumor suppressor and suppressed the proliferation and metastasis of various tumor cells such as adrenocortical carcinoma, colorectal cancer, hepatocellular carcinoma, and breast cancer cells [18, 19]. Wang et al. reported that miR-195-5p acted as the tumor suppressor in oral squamous cell carcinoma by interaction with TRIM14 gene [20]. Fei et al. [21] reported that miR-195-5p suppressed glucose uptake and proliferation of human BC cell line T24. In the present study, we found that miR-195-5p inhibitor rescued the effect of si-DLX6-AS1 on BC cells. It is noteworthy that miR-195-5p was down-regulated in BC tissues, and the *in vitro* results suggested that DLX6-AS1 negatively regulated the miR-195-5p expression. Based on the above findings, we demonstrated that DLX6-AS1 acted as an endogenous sponge RNA to interact with miR-195-5p in BC.

We further predicted and justified that VEGFA, is the target for miR-195-5p [22]. VEGFA is considered to be one of the important drivers of cancer angiogenesis and reported to related with increasing of vascular distribution, metastasis and leading to the poor prognosis of some cancers [22]. Studies investigate that the Ras/Raf/MEK/ERK signaling cascade plays a key role in the VEGF-mediated angiogenic signaling pathway for signaling and promoting VEGF expression [23]. Gu et al. investigated that Ras/Raf/Mek/Erk pathway was involved in the SHP2-induced growth and invaison of laryngeal cancer cells [24]. Inhibition of Ras/Raf/MEK/ERK signaling showed anti-tumor effect in cancers including BC [25]. In our study, we showed that VEGFA knockdown caused by DLX6-AS1 knockdown inhibited the phosphorylations of Raf-1, MEK1/2, and ERK1/2, while the miR-195-5p inhibitor or the VEGFA overexpression counteracted this eeffect. These results indicates that DLX6-AS1 accelerates cell proliferation, migration, and invasion of BC cells through targeting miR-195-5p and mediating VEGFA/Ras/Raf/MEK/ERK signaling pathway.

In conclusion, the present study demonstrates that DLX6-AS1 is up-regulated in BC tissues and cell lines. Knockdown of DLX6-AS1 shows tumor inhibition effect, which can be reversed by miR-195-5p inhibitor or VEGFA overexpression. However, the biological function and the definite mechanism of miR-195-5p and VEGFA in BC progression need to be further studied. These findings suggest that DLX6-AS1 functions as an oncogenic role in the development of BC through miR-195-5p-mediated VEGFA/Ras/Raf/MEK/ERK signaling pathway. DLX6-AS1 might be more a promising new target for BC prognosis and treatment in the future.

## MATERIALS AND METHODS

### Cell lines and animals

The human bladder epithelium immortalized cell line SV-HUC-1 and BC cell lines T24, RT4, 5637, J82, and SW780 were purchased from ATCC (MD, USA). The cells were cultured in DMEM (Gibco; Thermo Fisher Scientifc, Inc., Waltham, MA, USA) containing 10% fetal bovine serum (FBS; Invitrogen; Thermo Fisher Scientifc, Inc.) in an incubator with 5% CO_2_ at 37 °C.

Babl/c mice (20-24 g, 6 weeks) were purchased from Beijing HFK Bioscience Co. Ltd. (Beijing, China). The animals were housed in a laboratory with temperature control of 24 ± 2 °C and a humidity of 40%-60%. The animal experiment was approved by the medical ethics committee of The Affiliated Huaian No.1 People’s Hospital of Nanjing Medical University.

### BC tissue sample

Sixty clinical BC tissues and paired paracancerous tissues were from the resected specimens of BC patients from January 2006 to May 2012 in the Affiliated Huaian No.1 People’s Hospital of Nanjing Medical University (Huaian, China). The samples were confirmed pathologically by two pathologists independently and stored in liquid nitrogen. This study was approved by the Institutional Ethical Review Committee of the Affiliated Huaian No.1 People’s Hospital of Nanjing Medical University. All patients enrolled in the study signed the written informed consent.

### In situ hybridization (ISH)

The thickness of the tumor and normal tissue section was 4 μm, which was treated with 20 ug/mL protease K at 37 °C for 8 min post dewaxing. The section was pre-hybridized with ISH buffer (Exiqon), followed by hybridization with digoxigenin labelled probe for 40 min at 45 °C. Hybridized sections were incubated overnight with digoxigenin antibody (Roche Diagnostics IN) at 4°C and then were stained with nitroblue tetrazole/5-bromo-4-chloro-3- indolyl phosphate.

### Fluorescence in situ hybridization (FISH)

A FISH kit was purchased from Guangzhou Boye Biological Technology Co.,Ltd. (Guangzhou, China) and fluorescein isothiocyanate (FITC)-conjugated DLX6-AS1 DNA probe () was used for RNA-FISH. Cells were fixed in 4% formalin for 15 min. After prehybridization in PBS, the cells were hybridized at 37 °C for 30 min in hybridization solution. DAPI staining was performed and observed with a FV1000 confocal laser microscope (Olympus Corporation, Tokyo, Japan).

### Cell transfection

Over-expression of DLX6-AS1 and VEGFA were achieved by using a pcDNA3.1 vector (GenePharma Co., Ltd., Shanghai, China) transfection. The shRNA against DLX6-AS1 (5'-GTGATTCATACATCCCTATGG-3') was synthesized to knockdown of DLX6-AS1 (GenePharma) according to the BLOCK-iT™ RNAi Designer (Thermo). The miR-195-5p mimics and miR-195-5p inhibitor were designed and synthesized by Gene-Pharma. The empty vectors were used as control. The transfection was conducted by using Lipofectamine^TM^ 2000 (Invitrogen) in 5 × 10^6^ cells at a final concentration of 50 nM.

### Cell proliferation

Cell proliferation was measured using the CCK-8 kit (Beyotime, Shanghai, China). Briefly, after transfection, the cells were digested with pancreatin (Gibco) and collected, washed with PBS, and then resuspended in DMEM medium (Gibco) to 5×10^4^ cells/ml. The cell suspensions were added to a 96-well culture plate at 100 μl/well. The cells were incubated for 24, 48 and 72 h, respectively, and then 10 μl of CCK-8 solution was added to each well and incubated for 2 h. The absorbance was determined at 450 nm using an Infinite M200 spectrophotometer (TECAN). Six duplicates for each group.

### TUNEL

Cell apoptosis was examined by terminal deoxynucleotidyl transferase-mediated deoxyuridine triphosphate nick end labeling (TUNEL) (Roche Diagnostics, Mannheim, Germany). Briefly, after transfection, the cells were digested and collected, washed with PBS, and then resuspended in DMEM medium to 5×10^4^ cells/ml. The cell suspensions were added to a 24-well culture plate at 500 μl/well. The cells were incubated for 24 h. The cells were washed with PBS and then fixed with 4% paraformaldehyde for 30 min. After washing with PBS once again, the PBS containing 0.3% Triton X-100 was added and incubated for 5 min at room temperature. Then the cells were washed with PBS twice and 50 μl of TUNEL assay solution (prepared according to the instructions) was added and incubated at 37 ° C for 60 min in the dark. The cells were washed with PBS three times and sealed with the antifade mounting medium and observed under a fluorescence microscope (EX: 450-500 nm, EM: 515-565 nm; Olympus Corporation, Tokyo, Japan).

### Migration assay

The migration activity of BC cells was evaluated by wound healing assay. Briefly, after transfection, the cells were digested and collected, washed with PBS, and then resuspended in DMEM medium to 1×10^6^ cells/ml. The cell suspensions were added to a 6-well culture plate at 1 ml/well. After the cells were covered with the bottom of the plate, the cell layers were scratched by a perpendicular tip to form a wounded gap, and the scraped cells were washed away with PBS. The serum-free medium was added and incubated for 24 h. Finally, the gap width was measured.

### Invasion assay

The invasion activity of BC cells was determined by the Transwell chamber assay. First, the matrigel was diluted to 1 mg/ml and 100 μl diluted matrigel was added to the upper chamber. Then, after transfection, the cells were digested and collected, washed with PBS, and then resuspended in serum-free DMEM to 1×10^4^ cells/ml. Then 400 μl cell suspension was added to the upper chamber and 1 ml DMEM containing 10% serum was added to the lower chamber. The cells in the chamber were incubated in an incubator for 24 h, then the cells were fixed with methanol for 20 min, and stained with crystal violet for 20 min. Finally, we examined the stained cells under a microscope (Olympus Corporation), and assessed them in five random fields, and calculated the average number.

### RNA isolation and qRT-PCR

The total RNAs of BC tissues and cells were extracted with Trizol reagent (Invitrogen), the total miRNAs were extracted with miRcute miRNA isolation kit (Genconway) according to the manufacturer's instructions. For miRNA assay, the reverse transcription of the first strand of cDNA was performed by miRNA first-strand cDNA synthesis kit (TIANGEN). Briefly, the poly A tail was added to the 3' end of miR-195-5p, and then the Poly(A) modified miR-195-5p was subjected to reverse transcription to generate the first strand of cDNA according to the manufacturer's instructions. The qRT-PCR assay of the miR-195-5p cDNA was performed by miRcute miRNA fluorescence quantification kit (TIANGEN). Reaction system: 20 μl, reaction conditions: 94 °C for 2 min, (94 °C for 20 s, 60 °C for 30 s, 72 °C for 30 s) × 45 cycle. U6 was used as an internal control. For mRNA assay, the reverse transcription of the first strand of cDNA was performed by iScript cDNA Synthesis kit (Bio-Rad) according to the manufacturer's instructions. Then the cDNAs were subjected to qRT-PCR assay with an SYBR Green Realtime PCR kit (TOYOBO). Reaction system: SYBR Green Realtime PCR Master Mix 7.5 μl, upstream and downstream primers each 0.25 μl, RT product 1 μl, and PCR water supplemented to 15 μl. Reaction conditions: 94 °C for 2 min, (94 °C for 15 s, 55 °C for 30 s, 72 °C for 30 s) × 45 cycles. GAPDH was used as an internal control. The relative expression levels were calculated using 2^-ΔΔCT^ method [26].

### Western blot

The BC tissues or cells were lysed with pre-cooled RIPA lysate (Sigma-Aldrich) and protease inhibitor cocktail (Beyotime). Then the tissue or cells were homogenized on ice, and the homogenates centrifuged at 12 000 rpm for 10 min at 4 °C. The supernatants were collected and the protein concentration was measured by the BCA method. Then the loading buffer was added to the proteins and boiled for 10 min in a water bath. The proteins were subjected to SDS-PAGE gel electrophoresis at 20 μg/well, and then the proteins were transferred to a PVDF membrane. The transferred PVDF membrane was washed in TBST for 5 min and blocked with 5% skim milk for 2 h at room temperature. Then the PVDF membrane was placed in a hybridization bag and probed with primary anti-VEGFA (#ab1316, Abcam), anti-Ras (#ab16907, Abcam), anti**-**phospho-Raf-1 (#ab173539, Abcam), anti-Raf-1 (#ab50858, Abcam), anti-phospho-ERK1/2 (#ab214362, Abcam), ERK1/2 (#ab54230, Abcam), anti-phospho-MEK1/2 (#2338, Cell Signaling Technology), anti-MEK1/2 (#4694, Cell Signaling Technology), and GAPDH (#ab8245, Abcam) antibody that diluted with the blocking solution were added and incubated at 4 °C overnight. The PVDF membrane was washed with TBST buffer three times for 10 min each time, then the secondary antibody diluted in blocking solution (HRP goat anti-mouse) was added and incubated at 37 °C for 1.5 h. The immunoreactive protein bands were visualized by chemiluminescence using an Amersham prime ECL Plus detection system (GE Healthcare Life Sciences).

### Subcellular fractionation assay

The cytoplasmic and nuclear were extracted from BC cells using the PARIS™ Kit (Invitrogen). The extracts of cytoplasmic and nuclear were subjected to qRT-PCR assay. U6 as nuclear control, GAPDH as the cytoplasmic control.

### Dual-luciferase reporter assay

**T**he binding was determined by dual-luciferase reporter assay system (Promega). The target sequences were amplified by PCR and inserted into the pmirGLO vector (Promega) to construct the luciferase reporter vectors (pmirGLO-DLX6-AS1-wt, pmirGLO-DLX6-AS1-mut, pmirGLO-VEGFA-wt, pmirGLO-VEGFA-mut). Then these reporter vectors were co-transfected with or without miR-195-5p mimics (NC mimics as a control) into HEK-293T cells, respectively. The luciferase activity was determined on an illuminometer (Berthold, Germany).

### RNA pull-down assay

Firstly, the RNA was biotinylated with Biotin RNA Labeling Mix (Roche) and transcribed with T7 polymerase (Promega). Then the DNA was digested with RNase-free DNase I (Promega) and purified by RNeasy Mini Kit (QIAGEN). Three μg of biotinylated RNA was added to RNA structure buffer (10 mM Tris pH 7.0, 0.1 M KCl, 10 mM MgCl_2_) and treated at 90 °C for 2 min, then placed on ice for 2 min, and stood at room temperature for 30 min. The T24 or RT4 cells (1×10^7^ cells) were suspended in 2 ml of PBS, then 2 ml of RIPA lysis buffer was added, and the cells were lysed for 1 h at 4 °C. The lysate was centrifuged at 12 000 g for 10 min at 4 ° C, and the supernatant was collected and transferred to an RNase-free tube. Then 400 ng biotinylated RNA and 500 μl RIP buffer were added to the tube and incubated for 1 h, then 50 μl of streptavidin agarose beads were added and incubated for 1 h. The beads were washed with RIP buffer 5 times and washing solutions were collected for RT-PCR or western blot assay.

### RIP assay

RIP assay was conducted in T24 and RT4 cells using Magna RIP RNA-binding protein immunoprecipitation kit (Millipore). Briefly, the cells were collected after washing with cold PBS and the RIP lysis buffer was added. Then the suspension was centrifugated and 100 μl cell lysates were transferred to the RIP immunoprecipitation buffer which contained Ago2-conjugated magnetic beads, the mouse IgG as a negative control (Millipore, MA, USA). The magnetic beads washed with RIP wash buffer and then incubated with proteinase K for 30 min at 55°C. The RNA was extracted for qRT-PCR assay.

### Tumor growth in vivo

Balb/c mice were depilated on the back, and then 100 μl of T24 and RT4 cells successfully transfected with sh-DLX6-AS1 or shNC were subcutaneously injected into the left dorsal (1×10^7^ cells/mouse). The tumor volumes were monitored weekly for 5 weeks. On day 35, the mice were sacrificed and the tumors were excised and weighted. The expressions of Ki67 and VEGFA were examined by immunohistochemistry (IHC) assay.

### Statistical assay

All data were expressed as means ± SD. Student’s t test, One-way ANOVA, Kaplan-Meier’s method, and the log-rank test were performed by SPSS 13.0. All data from at least three independent experiments. Statistical significance was accepted at *P* values *<* 0.05.

## Supplementary Material

Supplementary Figure 1
