# Supplementary material for: Long non-coding RNA DLX6-AS1 facilitates bladder cancer progression through modulating miR-195-5p/VEGFA signaling pathway
Source: Aging (Albany NY). 2020 Aug 3;12(16):16021–34. doi: 10.18632/aging.103374 (PMC7485696; doi:10.18632/aging.103374)
Supplement: Supplementary Figure 1 [file aging-12-103374-s001..pdf]

SUPPLEMENTARY FIGURE

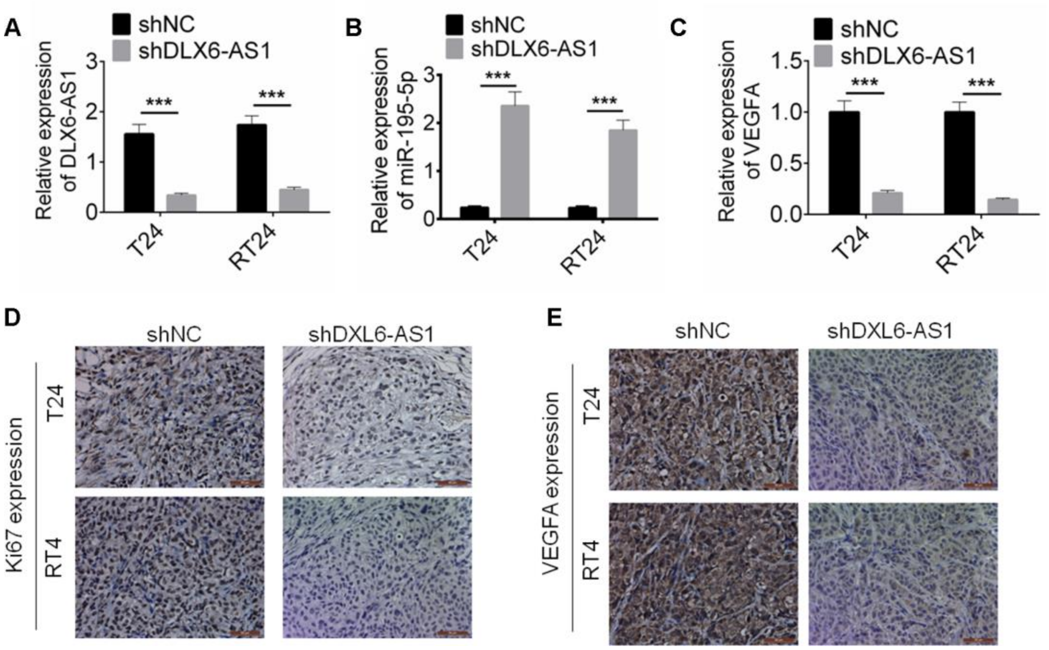

Supplementary Figure 1. shDLX6-AS1 promotes miR-195-5p expression and inhibits VEGFA expression *in vivo*. (**A**–**C**) The expressions of DLX6-AS1, miR-195-5p and VEGFA in the tumor tissues were examined by qRT-PCR. (**D**, **E**) The protein expressions of Ki67 and VEGFA in in the tumor tissues were examined by IHC analysis. Data were expressed as the mean ± SD. ***P < 0.001.
